# HoBi‐Like Pestivirus Is the Most Frequently Detected Pestivirus in Cattle Across Different Regions of Brazil

**DOI:** 10.1155/tbed/9404775

**Published:** 2026-07-06

**Authors:** Leticia F. Baumbach, Laura J. Camargo, Raquel S. Alves, Eduardo O. Sanguinet, Matheus O. Silva, Daniela T. Reis, Vitória Rabaioli, Gabriela E. Birlem, Roberto Schroeder, Fabiano Barreto, João Marcos N. Costa, Sara Hartke, Matheus N. Weber, Cláudio W. Canal

**Affiliations:** ^1^ Veterinary Virology Laboratory, Faculty of Veterinary Medicine, Federal University of Rio Grande do Sul (UFRGS), Porto Alegre, Rio Grande do Sul, Brazil, ufrgs.br; ^2^ Immunology and Molecular Biology Laboratory, Faculty of Veterinary Medicine, Federal University of Rio Grande do Sul (UFRGS), Porto Alegre, Rio Grande do Sul, Brazil, ufrgs.br; ^3^ Federal Agricultural Defense Laboratory (LFDA/RS), Ministry of Agriculture and Livestock (MAPA), Porto Alegre, Rio Grande do Sul, Brazil; ^4^ Secretariat of Agricultural Defense, Ministry of Agriculture and Livestock (MAPA), Brasília, Distrito Federal, Brazil; ^5^ Multiuser Molecular Biology Laboratory, Institute of Basic Health Sciences (ICBS), Federal University of Rio Grande do Sul (UFRGS), Porto Alegre, Rio Grande do Sul, Brazil, ufrgs.br

**Keywords:** BVDV, genetic diversity, genomic surveillance, HoBi-like pestivirus, molecular detection, phylogenetic analysis

## Abstract

Pestiviruses are major viral pathogens of cattle worldwide, causing substantial economic losses and posing persistent challenges to animal health and productivity. Brazil hosts the world’s largest cattle population and represents a strategic setting for the emergence and maintenance of bovine pestiviruses. Although previous studies have investigated pestiviruses in Northern, Northeastern, and extreme Southern Brazil, available molecular data remain geographically fragmented across this country of continental dimensions. HoBi‐like pestivirus, HoBiPeV (HoBiPeV) has been frequently detected in Northern and Northeastern regions in previous molecular studies, whereas BVDV‐1 and BVDV‐2 are more frequently detected in the extreme South, leaving large areas with limited molecular information. This study investigated the occurrence and genetic diversity of pestiviruses in cattle from Northern Brazil and Paraná state, a key region connecting Southern and Midwestern Brazil. A total of 20,267 bovine serum samples were screened by RT‐qPCR, including 10,832 from the Northern region (Acre, Amazonas, Rondônia, and Roraima) and 9,435 from Paraná State. Positive samples were subjected to sequencing and phylogenetic analyses. Pestivirus RNA was detected in 0.10% of samples (21/20,267), with HoBiPeV accounting for 90.5% of all detections. Notably, HoBiPeV predominated in both regions, reaching 93.3% of detections in Paraná, a state previously lacking molecular surveillance data. This study provides large‐scale molecular evidence supporting the circulation of HoBiPeV across distinct cattle‐producing regions of Brazil, where it was the most frequently detected pestivirus among the positive samples. These findings highlight the risk of silent HoBiPeV circulation that may escape BVDV diagnostic surveillance, which may facilitate undetected regional and potential undetected regional transboundary spread and pose a challenge to pestivirus control and eradication strategies. They also underscore the need for updated diagnostic approaches and continued genomic surveillance, as HoBiPeV circulation may be underestimated in South America and other cattle‐producing regions.

## 1. Introduction

Pestiviruses are widely distributed among cattle populations worldwide and, in the absence of coordinated control programs, remain endemic in most livestock‐producing countries [1]. Infections cause major reproductive and productive losses, including abortion, neonatal mortality, reduced milk yield, and decreased weight gain [2]. Economic losses per infected animal may reach USD 687.80, making bovine viral diarrhea (BVD) one of the most economically impactful viral diseases in cattle production systems [3]. Despite their recognized importance, Brazil, home to the world’s largest cattle population, still does not prioritize a national pestivirus control or eradication program, which contributes to the continued circulation of these viruses [4].

Members of the genus *Pestivirus* (family *Flaviviridae*) have a positive‐sense, single‐stranded RNA genome of ~12.3 kb that encodes a single polyprotein, which is cleaved by viral and cellular proteases into structural (C, Erns, E1, and E2) and nonstructural (N^pro^, NS2‐3, NS4A, NS4B, NS5A, and NS5B) proteins. The viral genome is flanked by untranslated regions (UTRs) at the 5′ and 3′ ends [5]. The 5′UTR is highly conserved and widely used for diagnosis purposes and species identification, whereas the *E2* gene, which encodes the major envelope glycoprotein, is more variable and provides greater discriminatory power for genotype and subgenotype classification [6].

The genus currently includes several species of veterinary relevance, such as *Pestivirus bovis* (BVD virus 1; BVDV‐1), *Pestivirus tauri* (BVDV‐2), and *Pestivirus brazilense* (HoBi‐like pestivirus, HoBiPeV) [7]. Based on recent evolutionary and taxonomic evidence, the establishment of *Pestiviridae* as a separate family has been proposed, replacing the current classification of *Pestivirus* as a genus within the *Flaviviridae*. Despite these proposals, the ICTV has not yet officially incorporated this change into its current taxonomy [8].

HoBiPeV shares many pathogenic features with classical BVDV species, including the ability to generate persistently infected (PI) calves, which are key drivers of viral maintenance [9, 10]. However, its marked genetic and antigenic divergence poses important challenges for the diagnosis, surveillance, and control. Several studies have demonstrated reduced sensitivity of commonly used molecular and serological assays when detecting HoBiPeV [11]. This divergence also results in limited cross‐neutralization, and BVDV vaccines have been shown to be ineffective in preventing HoBiPeV infection or fetal transmission [12]. Because most routine diagnostic assays and surveillance programs are designed for BVDV‐1 and BVDV‐2, HoBiPeV circulation may remain largely undetected, favoring its silent maintenance and spread within and between cattle populations.

Currently, no specific vaccine is available for HoBiPeV, and the antigenic divergence among pestivirus species results in low levels of cross‐neutralization [13]. Consequently, BVDV vaccines do not provide effective protection against HoBiPeV infection and continue to be used in the field despite their limited efficacy [14]. In regions where HoBiPeV predominates, as observed across multiple Brazilian territories, these limitations highlight the urgent need for diagnostic assays and vaccines that include HoBiPeV antigens.

Brazil hosts the world’s largest cattle population, estimated at over 238 million heads [15], and pestiviruses are considered endemic throughout the country [4]. Given Brazil’s continental dimensions, marked regional heterogeneity in pestivirus circulation is expected. Indeed, several studies have reported the wide circulation and genetic diversity of BVDV‐1 and BVDV‐2 in Brazilian herds [16], with marked regional variation. BVDV‐1a and BVDV‐2b predominate in the extreme southern region [17], whereas HoBiPeV is more frequently detected in the northern and northeastern regions [18, 19]. This heterogeneous pattern underscores the importance of genomic surveillance in Brazil, while the lack of molecular data across large central regions leaves significant gaps in understanding national pestivirus dynamics and may allow pestiviruses with limited surveillance coverage to circulate unnoticed.

Understanding the epidemiological behavior and genetic diversity of HoBiPeV is crucial for improving diagnostic tools, guiding vaccine strategies, and informing future control programs. Therefore, this study aimed to investigate the occurrence and molecular diversity of bovine pestiviruses in cattle from Northern and Southern Brazil using RT‐qPCR screening, sequencing, and phylogenetic analyses of partial 5′UTR and E2 regions, addressing critical gaps in molecular surveillance and providing robust molecular evidence of HoBiPeV circulation across distinct Brazilian regions.

## 2. Materials and Methods

### 2.1. Target Population and Sample Size

Serum samples analyzed in this study were collected between 2020 and 2021 as part of a national surveillance program coordinated by the Department of Animal Health (DSA/SDA) of the Brazilian Ministry of Agriculture and Livestock (MAPA). This surveillance is designed to demonstrate the absence of foot‐and‐mouth disease virus (FMDV) circulation in the national cattle population, in compliance with the standards established by the WOAH for maintaining Brazil’s FMD‐free status.

**Figure 1:**
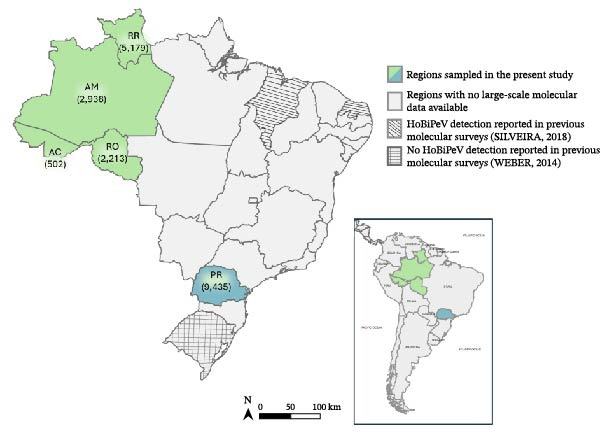
Geographic distribution of bovine serum samples analyzed in this study. Samples were collected from the states of Roraima (RR), Rondônia (RO), Amazonas (AM), Acre (AC), and Paraná (PR). The Northern region is shown in green, and Paraná State, located in the Southern, is highlighted in blue. The number of serum samples analyzed in each state is shown in parentheses. Solid colors indicate states sampled in the present study. Hatched areas indicate regions where HoBiPeV circulation has been previously reported [17, 18]. Grey areas represent regions with no large‐scale molecular surveillance data available. Detailed information on sampling locations is provided in Supporting Information 1: Table S1. The map was created using the free and open‐source geographic information system QGIS (version 3.4) [20].

A total of 20,267 bovine serum samples were included in the present investigation, originating from four states in the Northern region: Roraima (*n* = 5179), Rondônia (*n* = 2213), Amazonas (*n* = 2938), and Acre (*n* = 502) and from Paraná state (*n* = 9435) in Southern Brazil (Figure 1). Cattle aged between 6 and 24 months were randomly selected in each state [21]. All serum samples were stored at −80°C until analysis. The map was generated using the free and open‐source geographic information system QGIS (version 3.4) [20]. In Figure 1, solid‐colored states correspond to samples analyzed in the present study, whereas hatched regions summarize HoBiPeV molecular findings reported in previous studies [17, 18].

### 2.2. RNA Isolation and RT‐qPCR

Serum samples were initially screened for pestivirus RNA using a stepwise pooling strategy. To optimize large‐scale molecular testing, samples were first organized into pools of 100 serum samples. Positive pools were subsequently retested in pools of 10, followed by individual testing of positive samples. This approach, performed according to the manufacturer’s recommendations, reduced reagent consumption and labor requirements [22].

Total viral RNA was purified from serum samples using the MagMAX CORE Nucleic Acid Purification Kit (Applied Biosystems, CA, USA) in combination with the KingFisher 96 Flex automated extraction platform (Thermo Fisher Scientific, MA, USA), following the manufacturer’s instructions. The magnetic bead‐based workflow allows high‐throughput nucleic acid purification and was therefore employed for processing the large number of samples included in this study.

Initial molecular screening was performed using the VetMAX BVDV 4ALL Kit (Thermo Fisher Scientific, MA, USA). Samples were analyzed in pools containing 100 sera, as recommended by the manufacturer. The assay is designed for the detection of the currently recognized bovine pestivirus species, including BVDV‐1, BVDV‐2, and HoBiPeV. The effect of sample pooling on the assay performance was evaluated through internal validation experiments. Both naturally infected field samples and serially diluted positive controls were analyzed, including BVDV‐1 (NADL), BVDV‐2 (SV253), and HoBiPeV isolates maintained in a cell culture. Viral RNA remained detectable in simulated pools representing dilutions up to 1:100, supporting the use of this strategy for large‐scale surveillance activities.

Positive pools identified during the first screening step were subsequently retested in pools of 10 samples using the VetMAX Gold PI Detection Kit (Thermo Fisher Scientific, MA, USA). Individual samples from each positive 10‐sample pool were subsequently tested to identify positive samples, which were then confirmed by conventional RT‐PCR. An internal positive control (IPC) supplied with the extraction system was included in each run to monitor the extraction performance and verify RNA recovery throughout the process.

Quality control measures included the incorporation of two negative controls (serum from a pestivirus‐negative animal and a no‐template control containing ultrapure water) together with two positive controls (positive serum sample and kit‐provided positive control) in every RT‐qPCR run performed on 96‐well plates. For confirmation and molecular characterization, individual samples from positive pools were subjected to conventional RT‐PCR targeting partial sequences of the 5′UTR and E2 regions of the pestivirus genome.

Reverse transcription reactions were carried out using the High‐Capacity cDNA Reverse Transcription Kit (Applied Biosystems, CA, USA). The resulting cDNA was subsequently used for amplification of the target regions with Platinum Taq DNA Polymerase (Invitrogen, CA, USA) according to the manufacturer’s recommendations. Primer sequences are listed in Table 1.

**Table 1:** Primer pairs used for conventional PCR targeting partial sequences of the pestivirus 5′UTR and E2 regions.

Primer pair	Target region	Target size (bp)	Sequence (5′ – 3′)	Reference
BP189‐389	5′UTR	201	F: AGTCGTCARTGGTTCGAC R: TCCATGTGCCATGTACA	[23]
SF3‐SR3	E2	320	F: AAATGGTTGTAGGCGAGGACT R: GATACCGGCCCCTCACTCTG	[24]

### 2.3. Sanger Sequencing and Phylogenetic Analysis

Genetic characterization of bovine pestiviruses was performed through sequencing of partial 5′UTR and E2 amplicons obtained from RT‐PCR‐positive samples using the primer pairs described in Table 1. PCR products were purified with the PureLink PCR Purification Kit (Thermo Fisher Scientific, MA, USA) according to the manufacturer’s instructions.

Sequencing reactions were carried out using the BigDye Terminator v3.1 Cycle Sequencing kit and analyzed on an ABI PRISM 3100 Genetic Analyzer (Thermo Fisher Scientific, MA, USA). Raw chromatograms were inspected, assembled, and edited using Geneious Prime v.2025.2.1 (Biomatters, Auckland, New Zealand). Nucleotide identity was determined using the BLASTn (https://blast.ncbi.nlm.nih.gov/Blast.cgi; accessed on 3 September 2025). Reference pestivirus sequences were retrieved from GenBank (https://www.ncbi.nlm.nih.gov/genbank/; accessed on 3 September 2025) for phylogenetic comparisons. Multiple sequence alignments were generated with Fast Fourier Transform (MAFFT v.7) [25] using default settings. Phylogenetic trees were inferred using the maximum likelihood approach implemented in the IQ‐TREE web server (http://iqtree.cibiv.univie.ac.at) [26]. The most appropriate nucleotide substitution model was selected using ModelFinder [27]. The general time‐reversible model with gamma distribution and invariant sites (GTR + G + I) was applied when indicated [28, 29]. Branch support was estimated to be with 1000 bootstrap replicates. The partial 5′UTR and E2 sequences obtained in this study were deposited in GenBank under accession numbers PX496675‐PX496695 (5′UTR) and PX495051‐PX495065 (E2).

## 3. Results

### 3.1. Molecular Detection

A total of 20,267 bovine serum samples collected between 2020 and 2021 from six Brazilian states were analyzed by RT‐qPCR for pestivirus detection. Overall, 21 samples (0.10%) tested positive for pestivirus RNA. Positive samples were identified in both northern and southern regions (Table 2). In the Northern region, six samples (0.05%) were positive, including three from Roraima (3/5179; 0.06%), one from Rondônia (1/2213; 0.05%), and two from Amazonas (2/2938; 0.07%). No positive samples were detected in Acre (0/502). In the Southern region, 15 samples from Paraná State (15/9435; 0.16%) tested positive.

**Table 2:** Geographic distribution of pestivirus positive samples detected in cattle from Northern and Southern Brazil.

Region	State	Samples tested	Positives samples (%)	Species detected
Northern	Roraima (RR)	5179	3 (0.06)	HoBiPeV
Northern	Rondônia (RO)	2213	1 (0.05)	HoBiPeV
Northern	Amazonas (AM)	2938	2 (0.07)	HoBiPeV, BVDV‐1d
Northern	Acre (AC)	502	0 (0.00)	Not detected
Southern	Paraná (PR)	9435	15 (0.16)	HoBiPeV, BVDV‐1a

### 3.2. Phylogenetic Analysis

Sequencing the 5′UTR and E2 regions showed that 19 of the 21 pestivirus‐positive samples corresponded to HoBiPeV, whereas two were identified as BVDV‐1a or BVDV‐1d. Sequences detected in Northern and Southern Brazil showed high nucleotide identity (94%–100%) with previously reported HoBiPeV isolates based on both genomic regions (5′UTR and E2).

Phylogenetic analysis based on the partial 5′UTR region (Figure 2A) demonstrated that all HoBiPeV sequences clustered within the HoBiPeV‐a clade, supported by high bootstrap values. The BVDV‐1a and BVDV‐1d sequences detected in Paraná and Roraima, respectively, grouped with reference sequences representative of these subgenotypes, confirming their genetic classification based on the 5^′-^UTR region. Analysis of the partial E2 region (Figure 2B) produced a similar topological pattern, further confirming the classification of all HoBiPeV strains as the same species.

Figure 2Maximum likelihood phylogenetic trees based on partial sequences of the 5′UTR (A) and E2 (B) regions of pestiviruses detected in cattle from Northern and Southern Brazil. Tree constructed from 182‐nt (A) and 320‐nt (B) multiple sequence alignments. Brazilian sequences generated in this study are indicated by circles (●). Bootstrap (*n* = 1000 replicates) values >70% are shown at the nodes.

**Figure:**
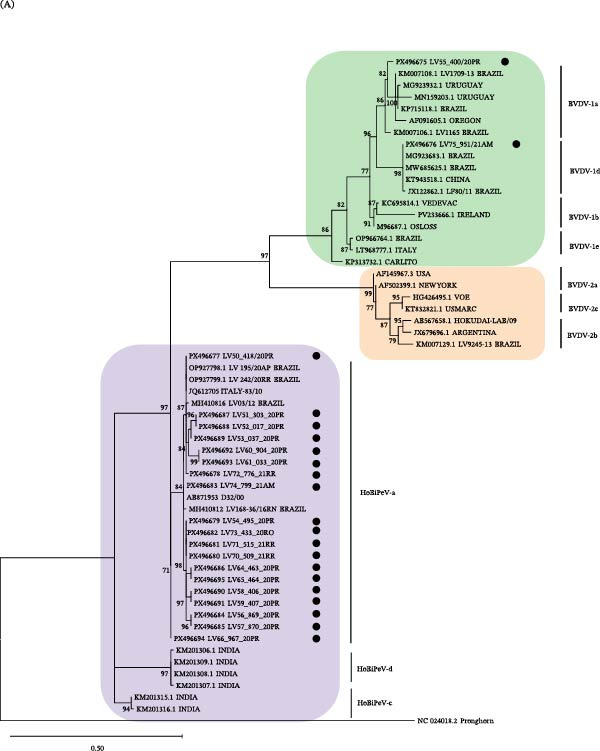

**Figure:**
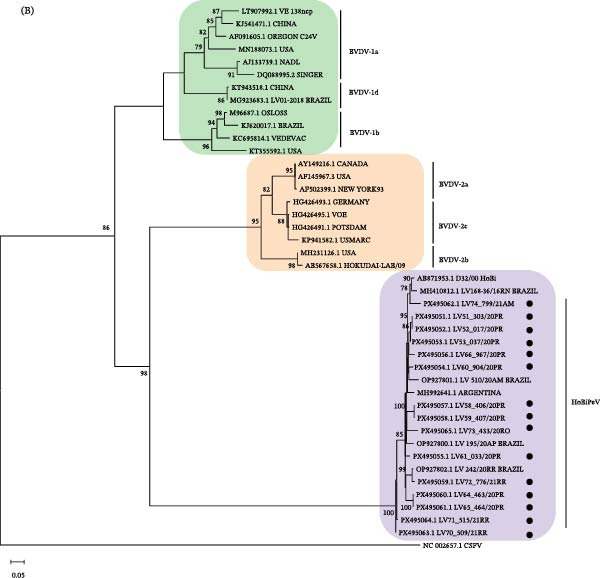

E2 amplification was successful for 15 of the 19 HoBiPeV‐positive samples, whereas amplification failed in the remaining samples, likely due to the high genetic variability and lower sequence conservation of this gene [30]. The E2 sequences obtained showed strong clustering with HoBiPeV isolates previously described in Brazil. The two BVDV‐1a and BVDV‐1d positive samples were not subjected to E2 amplification as the primers used were specifically designed for HoBiPeV detection [24]. To confirm their classification, these samples were further characterized by sequencing of the N^pro^ region, and their phylogenetic tree is shown in Supporting Information 2: Figure S1.

Together, these findings confirm that HoBiPeV was the frequently detected pestivirus species among the positive samples. The clustering of HoBiPeV sequences across both genomic regions, from northern and southern Brazil, demonstrates its widespread circulation among distinct cattle populations.

## 4. Discussion

This study provides the first large‐scale molecular evidence of HoBiPeV circulation across two contrasting cattle‐producing regions in Brazil. A total of 20,267 bovine serum samples were analyzed, and by integrating data from northern and southern regions, our findings extend previous regional reports and indicate that HoBiPeV circulation is not geographically restricted, occurring across distinct regions with diverse production systems and management contexts.

Although the overall detection frequency was low (0.10%), this finding is consistent with previous large‐scale molecular surveys conducted in Northern [19], Northeastern [18], and Southern Brazil [17] and reflects the silent epidemiological behavior of pestiviruses rather than indicating limited viral circulation. Most infections are transient and therefore rarely captured in cross‐sectional sampling, while PI animals play a key role in long‐term viral maintenance within cattle populations [31].

The age range of sampled animals (6–24 months), defined by the national surveillance program, may have influenced the frequency detection observed in this study. PI animals often have reduced survival beyond the first year of life, which may lead to underestimation of pestivirus detection, particularly among older animals [31]. In addition, the samples were obtained from a surveillance program primarily designed for FMDV monitoring rather than pestivirus epidemiology. While this standardized framework ensures random selection of herds and animals, broad geographic coverage, and consistency across regions, it may not be optimized for estimating the true prevalence of pestivirus infection. However, these factors are unlikely to affect the relative distribution of pestivirus species among the positive samples. Therefore, the predominance of HoBiPeV observed in this study remains a robust and consistent finding.

When integrated with previous molecular surveys (Figure 1), our results reveal a spatially continuous pattern of HoBiPeV circulation across Brazilian regions that have been molecularly characterized to date. These characterized areas surround large portions of the country where molecular surveillance data remain unavailable, suggesting that HoBiPeV may be circulating undetected across extensive geographic areas where molecular data are currently lacking.

In the Northern states, predominantly extensive grazing systems are characterized by low animal density and limited animal trade [32]. In contrast, Paraná, Brazil’s second‐largest milk‐producing state, is characterized by higher cattle density, intensive management practices, and greater animal movement [32], factors that may favor viral transmission and persistence within herds [33]. The detection of HoBiPeV in both extensive grazing systems in Northern Brazil and intensive dairy systems in Paraná indicates that viral maintenance is not restricted to a specific production model. Despite marked differences in animal density, management practices, and movement patterns, HoBiPeV circulation was confirmed in both contexts, highlighting its epidemiological adaptability. The high nucleotide identity (94%–100%) among HoBiPeV sequences from both regions, together with their consistent clustering within the HoBiPeV‐a clade, suggests the predominance of a well‐established viral lineage across different production contexts.

Historical records and molecular data suggest that HoBiPeV has been maintained in Brazil for several decades. Although the earliest identification occurred in 2004 in contaminated bovine serum [34], subsequent studies have documented widespread detection and considerable local diversity [18, 19], supporting long‐term endemicity rather than a recent introduction. Reports of genetically related strains in Argentina further suggest historical viral exchange within South America [35, 36], where livestock movement may facilitate silent transboundary dissemination.

Partial sequencing of the 5′UTR and E2 regions enabled consistent classification of the positive samples. The lower amplification success of the E2 region reflects its higher variability compared with the 5′UTR, a pattern reported in previous pestivirus studies [30] that highlights the need for molecular protocols specifically optimized for HoBiPeV.

A critical implication of our findings is that HoBiPeV circulation may remain largely undetected by routine surveillance systems. Most molecular diagnostic assays are designed for BVDV‐1 and BVDV‐2 and may show reduced sensitivity when detecting genetically divergent HoBiPeV strains, creating diagnostic blind spots that favor silent viral maintenance [14]. However, antigen detection assays, particularly those targeting the conserved Erns protein, have demonstrated broad reactivity across pestivirus species and may represent a valuable complementary diagnostic approach for the detection of HoBiPeV. Under these conditions, the combination of silent circulation and reduced molecular detection sensitivity creates favorable conditions for undetected transboundary spread. The detection of genetically related HoBiPeV strains in Brazil and neighboring South American countries suggests that animal movement and trade may facilitate viral dissemination across national borders without timely detection and reporting, particularly in regions relying primarily on BVDV‐focused molecular surveillance.

Currently available vaccines against bovine pestiviruses are typically based on BVDV‐1 and BVDV‐2 strains and include both modified live and inactivated formulations. In Brazil, most licensed vaccines are inactivated and incorporate a limited number of BVDV genotypes, most commonly BVDV‐1a, BVDV‐1b, and BVDV‐2a. The absence of vaccines specifically targeting HoBiPeV, combined with the limited cross‐neutralization induced by BVDV‐based formulations, likely contributes to its silent maintenance and its dissemination. Vaccines containing only BVDV strains show poor heterologous neutralization [14], and experimental evidence indicates that they fail to prevent fetal infection caused by HoBiPeV [37]. Consequently, in regions where HoBiPeV predominates, the continued use of vaccines containing only BVDV strains may inadvertently favor its persistence as the dominant pestivirus species [38].

The continued reliance on diagnostic assays and vaccines designed exclusively for BVDV‐1 and BVDV‐2, which show reduced sensitivity to HoBiPeV, represents a direct challenge to pestivirus control and eradication programs as such strategies may fail to detect or interrupt the circulation of an antigenically distinct virus, allowing HoBiPeV to persist and spread silently within and between regions [38]. This mismatch between circulating strains and vaccine composition may compromise current control strategies and contribute to the continued epidemiological success of HoBiPeV.

Although pooling strategies may reduce sensitivity for samples with low viral loads and potentially affect the detection of less abundant viral sequences, this approach is widely used in large‐scale pestivirus surveillance as a cost‐effective and efficient screening strategy [17, 22]. In the present study, pooling was combined with stepwise individual testing and internally validated conditions, supporting its suitability for large‐scale molecular screening. Nevertheless, the potential impact of pooling on analytical sensitivity should be considered when interpreting low‐frequency detection rates. Importantly, the low detection rate observed is consistent with other molecular surveys conducted in Brazil [17–19, 39], suggesting that the results are unlikely to be an artifact of the pooling strategy. Overall, these findings reinforce the relevance of structured molecular surveillance for understanding pestivirus circulation across distinct Brazilian regions.

Therefore, incorporating HoBiPeV into diagnostic assays, genomic surveillance strategies, and control programs is essential to mitigate its silent maintenance and transboundary dissemination. Strengthening molecular surveillance in regions with limited data and updating current diagnostic and immunization approaches are critical steps to mitigate the sanitary and economic impact of this emerging pestivirus at regional and cross‐border levels.

## 5. Conclusions

In summary, this large‐scale molecular survey confirms that HoBiPeV was the most frequently detected pestivirus among the analyzed samples. When integrated with previous findings from the Northern and northeastern regions [18, 19], the present results extend this pattern to Southern Brazil, indicating that HoBiPeV is silently maintained across distinct production systems and broad geographic areas. The detection of HoBiPeV in Paraná, a state with structured veterinary oversight, highlights its capacity to circulate under diverse sanitary conditions, suggesting that its distribution may be underestimated in regions with less systematic monitoring. Together, these findings have important sanitary and trade implications at regional and transboundary levels and reinforce the need to strengthen specific surveillance and to update diagnostic and immunization strategies to accurately detect and control this emerging pestivirus.

## Author Contributions

**Leticia F. Baumbach**: conceptualization, methodology, investigation, formal analysis, data curation, validation, writing – original draft, writing – review and editing. **Raquel S. Alves**, **Gabriela E. Birlem, and Laura J. Camargo**: investigation, data curation. **Eduardo O. Sanguinet**: investigation, writing – review and editing. **Matheus O. Silva and Sara Hartke**: investigation. **Daniela T. Reis and Vitória Rabaioli**: data curation. Roberto Schroeder, **Fabiano Barreto**, **João Marcos N. Costa**, **and Matheus N. Weber**: supervision, resources, writing – review and editing. **Cláudio W. Canal**: conceptualization, supervision, project administration, resources, Writing – review and editing.

## Funding

This work was supported by the Coordenação de Aperfeiçoamento de Pessoal de Nível Superior (CAPES) (Finance Code 001), the Conselho Nacional de Desenvolvimento Científico e Tecnológico (CNPq), the Fundação de Amparo à Pesquisa do Rio Grande do Sul (FAPERGS), and the Pró‐reitoria de Pesquisa da Universidade Federal do Rio Grande do Sul (Propesq/UFRGS).

## Ethics Statement

Ethical approval was not required for this study, as the bovine serum samples were previously collected through official surveillance programs conducted by the Brazilian Ministry of Agriculture, Livestock and Food Supply (MAPA) within the national Foot‐and‐Mouth Disease monitoring framework. No additional animal sampling or experimental procedures were performed for the purposes of this research. The samples were already available at the Veterinary Virology Laboratory of UFRGS and were authorized for research use under institutional regulations (Project Number 47831). Therefore, this study did not require approval from the Animal Ethics Committee (CEUA/UFRGS) or any additional ethical review.

## Conflicts of Interest

The authors declare no conflicts of interest.

## Supporting Information

Additional supporting information can be found online in the Supporting Information section.

## Supporting information


**Supporting Information 1** Table S1: Pestivirus screening results obtained from cattle sampled in Brazilian states between 2019 and 2021. Abbreviations: AC, Acre; AM, Amazonas; PR, Paraná; RO, Rondônia; RR, Roraima. Sex: F, female; M, male. Result: RT‐PCR detection of pestiviruses.

**Supporting Information 2** Figure S1: Neighbor‐joining phylogenetic tree based on partial Npro sequences of the two BVDV‐1 positive samples identified in this study. This analysis was performed for confirmatory purposes because amplification of the E2 region was unsuccessful for these samples due to the use of HoBiPeV‐specific primers. Sequences generated in this study are indicated by filled circles (●). Bootstrap values (>70%) obtained from 1000 replicates are shown at the nodes.

## Data Availability

The data that support the findings of this study are available in the supporting information of this article.
