# Supplementary figures and images for: HoBi‐Like Pestivirus Is the Most Frequently Detected Pestivirus in Cattle Across Different Regions of Brazil
Source: Transbound Emerg Dis. 2026 Jul 6;2026:9404775. doi: 10.1155/tbed/9404775 (PMC13334881; doi:10.1155/tbed/9404775)

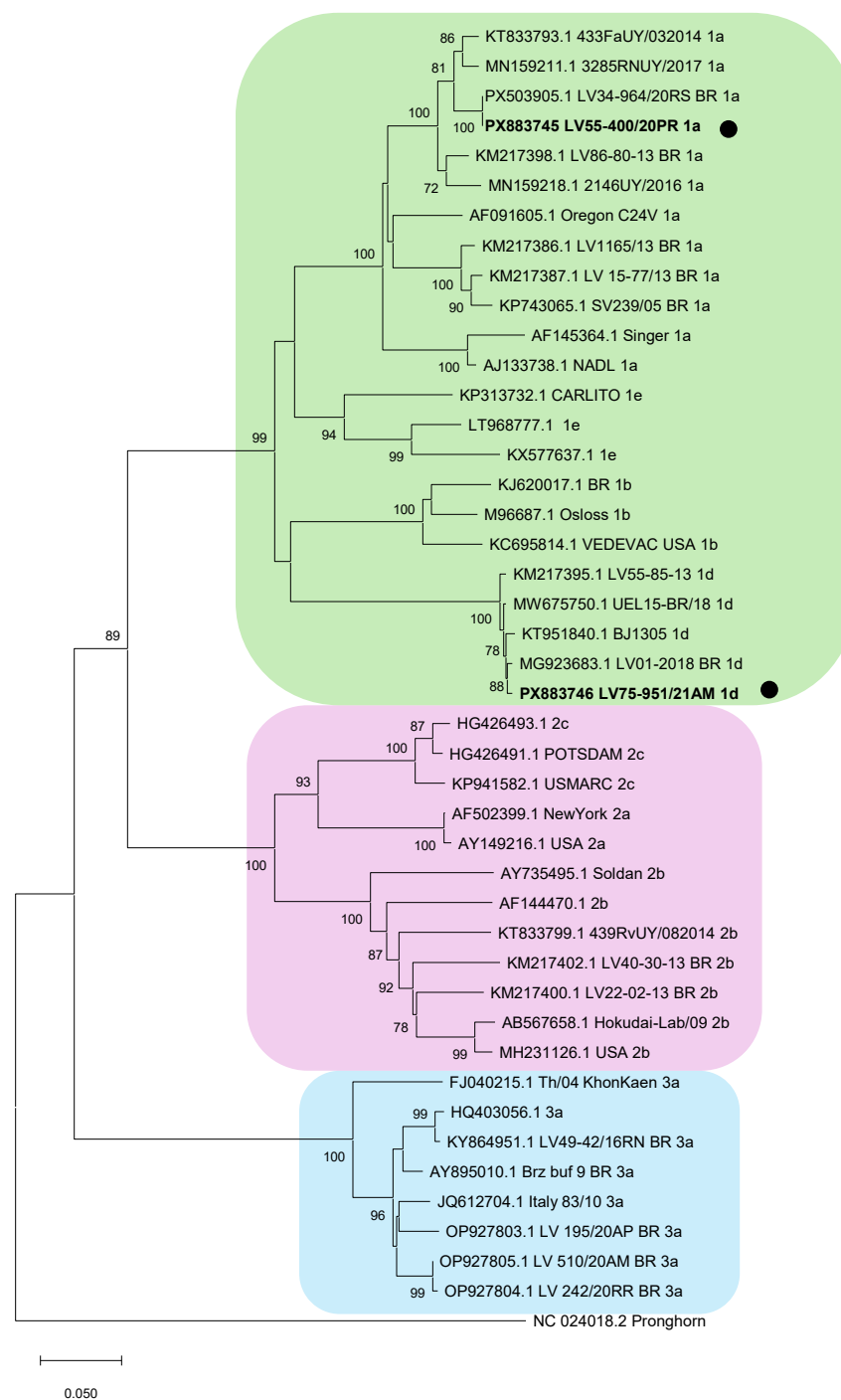

Supplement: Supplementary file 2 — Supporting Information 2 Figure S1: Neighbor‐joining phylogenetic tree based on partial Npro sequences of the two BVDV‐1 positive samples identified in this study. This analysis was performed for confirmatory purposes because amplification of the E2 region was unsuccessful for these samples due to the use of HoBiPeV‐specific primers. Sequences generated in this study are indicated by filled circles (●). Bootstrap values (>70%) obtained from 1000 replicates are shown at the nodes. [file TBED-2026-9404775-s001.pdf]
